# Uncovering the secrets of hidden twists

**DOI:** 10.1093/nsr/nwad215

**Published:** 2023-08-11

**Authors:** Oded Hod, Michael Urbakh

**Affiliations:** Department of Physical Chemistry, School of Chemistry, The Raymond and Beverly Sackler Faculty of Exact Sciences and The Sackler Center for Computational Molecular and Materials Science, Tel Aviv University, Israel; Department of Physical Chemistry, School of Chemistry, The Raymond and Beverly Sackler Faculty of Exact Sciences and The Sackler Center for Computational Molecular and Materials Science, Tel Aviv University, Israel

Twisted layered material junctions, and in particular twisted graphene interfaces, exhibit fascinating physical properties of diverse nature including unique structural, tribological, electronic, as well as heat and electron transport characteristics [1]. These properties are intimately related to the moiré superstructure formed at the twisted interface, which induces atomic lattice reconstruction patterns that balance between inter-layer registry optimization and intra-layer strain minimization [2]. When the twisted interface is embedded within a layered material stack, the interplay between intra- and inter-layer effects becomes even more intricate, as the twisted layer has to comply with constraints imposed by its two sandwiching adjacent layers. While the resulting structural deformation seeds at the twisted interface, it is not localized at that region and can propagate far inside the bulk [3,4]. Therefore, if the twisted interface is embedded not too deep into the stack, one may expect to observe its manifestation on the properties of the free surface. Hence, by characterizing the surface, one might be able to probe the properties of the underlying twisted interface.

**Figure 1.**
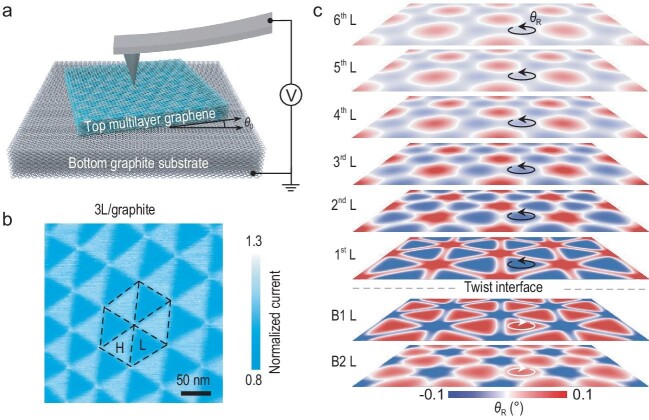
Experimental and force-field calculation results. (a) Schematics of the experimental setup. (b) A typical current map measured for the twisted 3 L/graphite sample. (c) Atomic deformation maps of various graphene layers within a multilayer stack obtained via classical force-field calculations. Adapted with permission from Ref. [5].

In the paper entitled ‘Deducing the internal interfaces of twisted multilayer graphene via moiré-regulated surface conductivity’ by the groups of Prof. Qunyang Li and Prof. Wengen Ouyang [5], the authors show that measuring the spatially resolved vertical electronic conductivity of a graphitic stack with a single stacking fault allows for the visualization of the reconstructed moiré superstructure of the embedded twisted interface. Using conductive atomic force microscopy techniques, the authors demonstrate that the small twist angle moiré superlattice regulates surface conductivity even if the twisted interface is located as much as 10 atomic layers beneath the surface (see Fig. 1).

The measurement results are rationalized by fully atomistic anisotropic force-field simulations, demonstrating the propagation of the intra-layer atomic reconstruction from the embedded twisted interface towards the stack surface. Based on these results, the authors construct a phenomenological classical electronic conductivity model that utilizes the registry index approach [6] to explain the measured surface conductivity maps. Notably, this simple treatment is able to reproduce the experimental results down to fine details, thus providing an explanation for the maximal conductivity contrast observed when the twisted interface appears two layers below the surface.

The innovative development of recent experimental microscopy techniques for characterizing twisted layered interfaces, suggests that intricate momentum space interference effects may dominate interlayer transport [7]. Interestingly, the fact that the experimental results presented by Li and Ouyang can be rationalized using simple classical considerations, indicates that the contribution of quantum interference effects is minor in this case, where local probing is utilized.

The joint experimental and theoretical tools developed by Li and Ouyang, thus pave the way for the non-invasive high resolution spatial characterization of twisted interfaces embedded within layered material stacks.
